# The Effectiveness of Social Support–Based Interventions for Dietary and Physical Activity in Adults Living With Overweight and Obesity: Protocol for a Systematic Review

**DOI:** 10.2196/81735

**Published:** 2025-12-23

**Authors:** Preeyapat Mangkalard, Abigail Fisher, Alexandra Burton, Dhikshita Chandana, Rana Conway

**Affiliations:** 1 Research Department of Behavioural Science and Health, University College London, 1-19 Torrington Place, London, WC1E 7HB, United Kingdom, 44 020 7679 1720; 2 Centre for Psychiatry and Mental Health, Queen Mary University of London, London, United Kingdom

**Keywords:** protocol, systematic review, meta-analysis, overweight, obesity, social support, intervention, diet, physical activity, PRISMA

## Abstract

**Background:**

Obesity is a global public health concern, affecting roughly 1 in 3 individuals. Social support has been identified as a key factor in promoting healthy behaviors, including dietary improvements and increased physical activity. While previous reviews have focused primarily on weight loss outcomes, there is limited evidence on how social support–based interventions specifically influence diet and physical activity behaviors.

**Objective:**

This study aims to evaluate (1) the effectiveness of social support–based interventions in improving dietary and physical activity behaviors among adults living with overweight and obesity (plus weight changes and psychosocial factors as secondary outcomes) and (2) identify which types of social support are most effective in facilitating positive behavioral changes.

**Methods:**

A comprehensive search will be conducted across 7 databases and registers (MEDLINE, Embase, PsycINFO, CINAHL, Web of Science, Cochrane Library, and ClinicalTrials.gov) and gray literature sources (Google Scholar, ProQuest, and PsyArXiv), in accordance with PRISMA (Preferred Reporting Items for Systematic Reviews and Meta-Analyses) guidelines. Two independent reviewers will screen randomized controlled trials and quasi-experimental studies involving adults living with overweight or obesity who have participated in social support–based interventions targeting diet and physical activity. The revised Joanna Briggs Institute critical appraisal tools will be used to assess the risk of bias. Findings will be reported qualitatively, and where appropriate, meta-analyses will be performed to generate pooled effect estimates for the primary outcomes. Secondary outcomes will also be reported qualitatively.

**Results:**

A pilot database search was conducted on November 29, 2024, to refine the search syntax. The screening, data extraction, quality assessment, and analysis will be conducted, with completion expected by March 2026.

**Conclusions:**

This review will synthesize current evidence on the effectiveness of social support–based interventions for improving diet and physical activity among adults living with overweight and obesity. The findings will help inform the development of future interventions and contribute to public health strategies.

## Introduction

Obesity is a chronic, complex disease characterized by disproportionate fat accumulation that worsens health. It is a worldwide public health concern. Over 43% of adults aged 18 years and older are overweight, and 16 % live with obesity [1]. At least 2.8 million people die each year as a consequence of being overweight or obese worldwide [1].

Excessive dietary energy intakes and insufficient physical activity are among the primary drivers of overweight and obesity [2]. Managing obesity requires a multisectoral approach that extends beyond individual responsibility, encompassing interpersonal support, community engagement, environmental modifications, and policy interventions [3]. Social support comprises efforts to aid individuals and to foster their sense of connection to meaningful social groups [4]. Social support covers multiple dimensions, including emotional, instrumental or tangible, informational, and appraisal support [5].

Social support is important for achieving positive changes in a range of risk factors for chronic diseases, such as overweight and obesity [6,7]. A longitudinal study highlighted that social support from friends and coworkers for healthy eating, and from family for physical activity, was associated with weight reduction, whereas family social undermining of healthy eating predicted weight gain among employed adults [8]. A recent systematic review identified 24 social support–based weight loss interventions lasting between 6 weeks and 18 months (median 3.5 mo) [9]. Meta-analyses indicated that these interventions resulted in uncertain effects when assessed during the intervention period, but significant benefits at longer-term follow-ups, particularly at the end of treatment and at 3- and 6-month postintervention assessments [9]. Another meta-analysis revealed that in 20 articles reporting on 15 online, group-based social support interventions for adults with severe obesity lasting between 3 and 18 months (median 6 mo), participants achieved significant weight loss immediately after treatment [10]. These reviews primarily assessed weight loss outcomes [9,10]. However, intervention effects on dietary and physical activity behaviors were inconsistently assessed, indicating a need for more comprehensive evaluation [10].

Systematic reviews of social support–related interventions for individuals with obesity and overweight have focused primarily on weight loss outcomes [9,10]. However, we were unable to identify a systematic review of evidence on the effectiveness of such interventions on improving dietary and physical activity behaviors in this population. Although weight is a key parameter for assessing obesity, these behaviors are the primary drivers of overweight and obesity [2]. Focusing on these behaviors provides a better understanding of how social support facilitates sustainable lifestyle change, which may contribute to weight management [11]. Therefore, we aim to conduct a systematic review of the effectiveness of social support–based interventions for improving diet and physical activity among adults living with overweight and obesity. Review questions include the following: (1) How effective are social support–based interventions in improving diet among adults living with overweight and obesity? (2) How effective are social support–based interventions in increasing physical activity among adults living with overweight and obesity? and (3) Which types of social support are most effective in promoting dietary and physical activity behavior change among adults living with overweight and obesity?

## Methods

To maintain project integrity, the review protocol was registered with PROSPERO (CRD42024598335) on November 22, 2024 [12], and the review will adhere to the PRISMA (Preferred Reporting Items for Systematic Reviews and Meta-Analyses) guidelines for its conduct and reporting [13].

### Inclusion and Exclusion Criteria

Inclusion and exclusion criteria were developed based on the PICOS (population, intervention, comparison, outcome, and study design) framework, as shown in Table 1. Only randomized controlled trials (RCTs) and quasi-experimental (QE) studies will be included to ensure methodological rigor and allow a more reliable assessment of intervention effects. Nonrandomized and before-and-after studies will be excluded, as the absence of control groups and randomization limits the ability to draw causal inferences about intervention effectiveness.

Operational criteria for screening will be applied to differentiate social support–based interventions from general lifestyle interventions as follows:

- Structured facilitation: the social support mechanism must be structured and facilitated by the intervention design. This includes interventions that provide specific platforms, schedules, or mechanisms for interaction (eg, assigned peer buddies, facilitated group discussions, or mandatory family attendance at sessions).
- Distinct component: social support must be identifiable as a distinct and active intervention component (eg, labeled as social support, peer-led, or family-based). To ensure clear operational boundaries and interrater reliability, we will classify types of social support based on the primary source of the support. We define these categories as family-based, peer-based, community-based, and mixed source. Details are included in Multimedia Appendix 1.

**Table 1.** Inclusion and exclusion criteria developed using the PICOS (population, intervention, comparison, outcome, and study design) framework.

	Inclusion criteria	Exclusion criteria
Population	Adults (aged >18 y) living with overweight or obesity	Adolescents (aged <18 y)
Intervention	Interventions based on type of social support [5,14]: Family-based support Community-based support Peer-based support Mixed source Intervention focus: Interventions focused on promoting healthy dietary behavior changes Interventions that aim to increase physical activity	Nonsocial support interventions: Studies focusing solely on individual-based interventions without any social support component Interventions providing only professional guidance or education
Comparator	Studies with any type of control condition will be included	Studies without comparator
Outcomes	Primary outcomes include measurement of any component of diet or physical activity, such as total calorie intake, daily fruit and vegetable intake, total walking score, or total energy expenditure Secondary outcomes may include changes in psychological variables, such as perceived social support, quality of life, or overall well-being, with corresponding measures of effect assessed similarly Interventions that clearly state duration and a measurable follow-up period to assess behavior change	Studies without a clear measure of diet or physical activity
Study design	Randomized controlled trials Quasi-experimental studies	Other study designs

### Searching and Screening

#### Electronic Searches

The search will be restricted to the following databases and registers: MEDLINE (Ovid), Embase (Ovid), PsycINFO (Ovid), CINAHL (EBSCO), Web of Science (Core Collection), Cochrane Library (Wiley), and ClinicalTrials.gov. Additionally, gray literature sources (Google Scholar, ProQuest, and PsyArXiv) will be searched. Only studies published in English, regardless of publication date, will be included. Retracted studies will be excluded.

#### Search Strategy

Search strategies were developed based on the PICO (population, intervention, comparison, and outcome) framework [15], as outlined in Table 2. The proposed search strategies for all databases are detailed in Multimedia Appendix 2.

**Table 2.** Search strategies.

Element	Terms
P (population)	obesity OR overweight OR “body mass index” OR BMI
I (intervention)	“social support” OR “peer support” OR family-based OR community-based
C (comparison)	usual care OR control OR “no intervention”
O (outcome)	diet* OR nutrition* OR “physical activity” OR exercise

#### Study Selection

Following the database search, duplicate records will be identified and removed. Two independent authors (PM and DC) will screen all titles and abstracts to assess studies for potential inclusion based on the predefined eligibility criteria. For studies deemed potentially relevant, full-text articles will be retrieved and independently assessed by the same reviewers to determine final eligibility.

Reasons for exclusion will be documented at the full-text stage, such as lack of dietary or physical activity outcomes, baseline-only studies without follow-up, feasibility studies, or protocol registrations.

Any disagreements between the 2 reviewers will be resolved through discussion. If consensus could not be reached, a third reviewer (RC) will be consulted.

The study selection process will be documented in sufficient detail to complete a PRISMA flow diagram.

Rayyan (Rayyan Systems, Inc) will be used to organize, screen, and manage all identified records from the database searches [16].

### Data Extraction and Management

A data extraction form will be created by PM in Microsoft Excel to capture study characteristics, intervention details, and outcome data. Data entry will be double-checked by comparing the extracted table with the original article reports. Information will be extracted from each included study, with categories provided in Multimedia Appendix 3.

If any data are unclear or missing, the corresponding author of the study will be contacted, with 1 follow-up email, to request additional information. If no response is received within 4 weeks, the specific data or outcome will be excluded from the analysis, with the reason for exclusion documented in the review. Where feasible, sensitivity analyses will be conducted to assess the potential impact of missing data on the overall findings.

### Quality Assessment

Two independent reviewers (PM and DC) will assess the risk of bias of each study using the Joanna Briggs Institute (JBI) critical appraisal tools, selected according to study design. These include the revised JBI tool for RCTs and QE studies. The revised JBI tool for RCTs evaluates 5 domains: bias related to selection and allocation; administration of the intervention and exposure; assessment, detection, and measurement of outcomes; participant retention; and statistical conclusion validity. The revised JBI tool for QE studies evaluates 7 domains: temporal precedence; selection and allocation; confounding factors; administration of the intervention and exposure; assessment, detection, and measurement of outcomes; participant retention; and statistical conclusion validity [17,18]. Each item will be rated as yes, no, unclear, or not applicable. An overall risk of bias judgment will be made for each study using the grading scale adapted from a previous systematic review, where studies with 3 or fewer “no” responses were classified as low risk, those with 4 to 6 “no” responses as moderate risk, and those with more than 6 “no” responses as high risk [19]. This approach was used since the original JBI tool does not provide explicit thresholds for determining the overall risk of bias. The Grading of Recommendations Assessment, Development, and Evaluation approach will not be applied, as this review does not aim to produce certainty of evidence ratings or guideline recommendations; instead, the focus is on evaluating methodological quality and synthesizing findings across study designs. Publication bias will be examined qualitatively when fewer than 10 studies are included and, where 10 or more studies are available, assessed formally using funnel plots and Egger regression test [20].

### Data Analysis and Statistical Analyses

Study characteristics, intervention details, and outcome data will be summarized in tabular form, accompanied by a narrative synthesis. Where data are qualitative or not suitable for statistical analysis, descriptive summaries will be provided. Functional dimensions of the support, including emotional, instrumental, informational, and appraisal, will be discussed qualitatively. If at least 5 studies provide comparable outcome data, a meta-analysis will be conducted to evaluate differences between intervention and control groups. This threshold was chosen to ensure stable estimation of between-study variance and to maintain adequate power for detecting heterogeneity, as recommended in previous methodological research [20,21]. For continuous outcomes, pooled results will be expressed as mean differences or standardized mean differences when measurements differ with 95% CIs, while dichotomous outcomes will be reported using relative risks with 95% CIs.

To address heterogeneity in the measurement of diet and physical activity, outcomes will be harmonized prior to meta-analysis. We will mathematically convert reported units to uniform metrics where feasible (eg, kcal to kJ, daily to weekly amounts, etc). However, incompatible outcomes will be kept as distinct domains (eg, separating total energy intake from diet quality scores). Where possible, the standardized mean difference will be used to pool effects within specific domains.

Heterogeneity will be assessed to examine the differences between studies using Cochran Q and the *I*² statistic, and results will be interpreted cautiously when fewer than 10 studies are available, as heterogeneity estimates and small-study effects are less reliable in small meta-analyses [22]. If substantial heterogeneity is detected (*I*²>50%), subgroup analyses will be conducted, or meta-regression analyses will be performed for continuous variables. A random-effects model will be applied throughout [23]. Sensitivity analyses will be carried out to assess the robustness of pooled results.

Meta-analysis and statistical analyses will be conducted using JASP (version 0.95.4).

## Results

**Figure 1.**
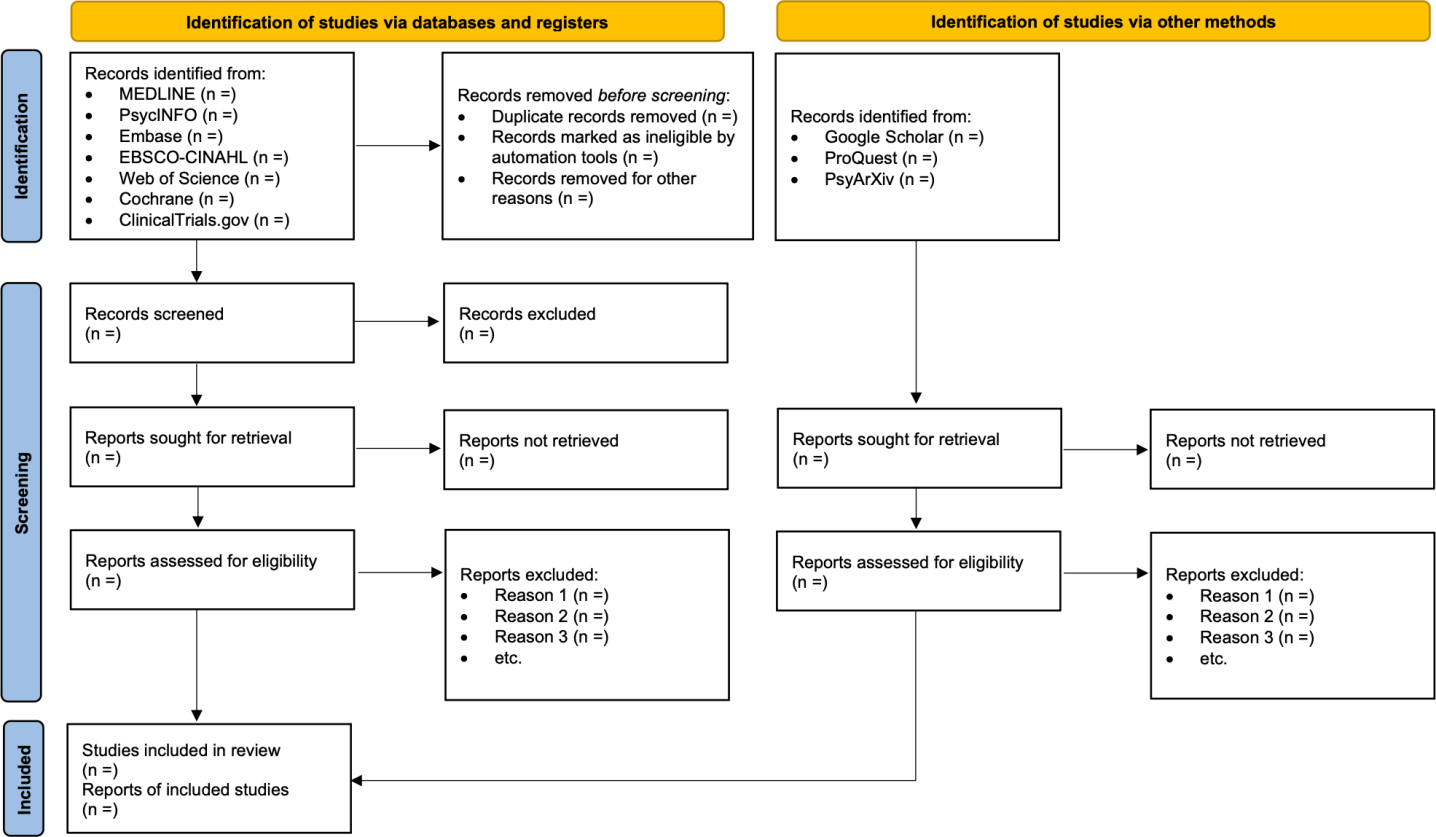
Preferred Reporting Items for Systematic Reviews and Meta-Analyses flow diagram template.

The project was funded in August 2024. A pilot database search was conducted on November 29, 2024, to refine the search syntax and confirm the suitability of selected keywords and indexing terms. This preliminary step informed the final search strategy that will be used for the full systematic review. This review will adhere to the PRISMA guidelines for its conduct and reporting as shown in Figure 1 [13].

No formal screening, data extraction, or analysis has yet been undertaken. The full systematic search and screening will be conducted in December 2025, with data extraction and synthesis expected to be completed by March 2026.

## Discussion

### Anticipated Findings

This review is a part of a broader study aimed at developing a social support–based intervention prototype focused on improving diet and physical activity among adults living with overweight and obesity. Subsequent stages of the project will involve patient and public involvement to shape the research direction, including qualitative interviews to explore the needs of the target population and co-design workshops to develop the intervention. Therefore, it is essential to review existing literature to identify effective interventions that incorporate social support elements for behavior change.

The findings from this review will enhance understanding of current evidence on social support–based interventions for overweight and obesity and help identify key research gaps to inform future development work. With obesity rates rising globally and dietary and physical activity behaviors remaining the major drivers of excess weight, evidence on effective support strategies is needed. This review will inform the creation of scalable, adaptable interventions that can promote behavior change worldwide.

### Strengths and Limitations

This review protocol has notable strengths. It adheres to rigorous methodological standards, including registration with PROSPERO and compliance with PRISMA guidelines, which ensures transparency and reproducibility. The comprehensive search strategy spans 6 major databases, 1 register, and 3 gray literature sources, increasing the likelihood of capturing a broad range of relevant studies. The inclusion criteria are clearly defined, focusing on structured social support interventions targeting both dietary and physical activity behaviors. The use of validated tools for risk of bias assessment, such as the revised JBI critical appraisal instruments, enhances the reliability of quality evaluations. Additionally, the dual-reviewer approach for screening and data extraction, with a third reviewer available to resolve disagreements, further strengthens the methodological rigor.

However, this review also has some limitations. First, the inclusion of only English language publications may introduce language bias and limit the comprehensiveness of the evidence base. Second, the variability in outcome measures, particularly in how diet and physical activity are assessed, may affect the consistency and interpretability of the results. Therefore, outcomes will be harmonized before meta-analysis. Finally, it is not possible to assess the intensity of the social support component of interventions (eg, percentage of contact time). However, preliminary exploration of the data found that this was not usually reported and was deemed an inappropriate indicator of intervention quality.

### Conclusions

This protocol will systematically review social support–based interventions for diet and physical activity among adults living with overweight and obesity to identify the most effective support mechanisms and key intervention characteristics. By integrating quantitative and qualitative evidence, it will uncover research gaps and inform the development of tailored, evidence-based public health strategies to reduce the global obesity burden. As previous reviews have not fully examined how different forms of social support affect dietary and activity outcomes in this population, our work will fill this gap and guide the design of more effective, socially informed interventions.

## Supplementary material

10.2196/81735 Multimedia Appendix 1 Operational definitions for social support types.

10.2196/81735 Multimedia Appendix 2 Proposed search strategies.

10.2196/81735 Multimedia Appendix 3 Data extraction categories.

10.2196/81735 Checklist 1 PRISMA-P checklist.

## Abbreviations

JBI: Joanna Briggs Institute
PICO: population, intervention, comparison, and outcome
PICOS: population, intervention, comparison, outcome, and study design
PRISMA: Preferred Reporting Items for Systematic Reviews and Meta-Analyses
PROSPERO: International Prospective Register of Systematic Reviews
QE: quasi-experimental
RCT: randomized controlled trial
