# Supplementary material for: The Effectiveness of Social Support–Based Interventions for Dietary and Physical Activity in Adults Living With Overweight and Obesity: Protocol for a Systematic Review
Source: JMIR Res Protoc. 2025 Dec 23;14:e81735. doi: 10.2196/81735 (PMC12726821; doi:10.2196/81735)
Supplement: Multimedia Appendix 1 [file resprot-v14-e81735-s001.docx]

**Multimedia Appendix 1.** Operational definitions for social support types.

1. Family-Based Support

- Definition: Interventions involving people that the participants already live with or has a close relationship with.
- Boundary: This includes anyone in the same household such as spouses, partners, parents, siblings, children, or other household members.
- Exclusion: Distant relatives who do not live with the participant and are not actively involved in their daily life.

2. Peer-Based Support

- Definition: Interventions involving people who share a something in common with the participant such as similar BMI, age range, gender, or health goal but are not close friends or family.
- Boundary: This can be buddies assigned by researchers, fellow participants in a group intervention, or mentors who are expert patients (e.g., someone who has successfully lost weight).
- Exclusion: Professional health coaches or counsellors are not peers.

3. Community-Based Support

- Definition: Interventions involving wider community such as local health workers, community health volunteers, or neighbourhood associations outside the immediate family.
- Boundary: This includes support delivered through community hubs such as faith-based churches, workplace networks, neighbourhood associations.
- Unlike peer-based support (which focuses on shared patient experience), community-based support focuses on supporting community infrastructure and culturally relevant local leaders.

4. Mixed/Multi-Source Support

- Interventions that clearly combine two or more of the above components (e.g., a study involving both spousal participation and peer group meetings) will be defined as mixed support.
