# Supplementary material for: The Effectiveness of Social Support–Based Interventions for Dietary and Physical Activity in Adults Living With Overweight and Obesity: Protocol for a Systematic Review
Source: JMIR Res Protoc. 2025 Dec 23;14:e81735. doi: 10.2196/81735 (PMC12726821; doi:10.2196/81735)
Supplement: Multimedia Appendix 2 [file resprot-v14-e81735-s002.docx]

**Multimedia Appendix 2.** Proposed search strategies

**Table S1.** Proposed search strategy for MEDLINE (Ovid)

| Order | Strategy |
| --- | --- |
| 1 | (social support* intervention or program*).mp. [mp=title, book title, abstract, original title, name of substance word, subject heading word, floating sub-heading word, keyword heading word, organism supplementary concept word, protocol supplementary concept word, rare disease supplementary concept word, unique identifier, synonyms, population supplementary concept word, anatomy supplementary concept word] |
| 2 | behavio* change.mp. [mp=title, book title, abstract, original title, name of substance word, subject heading word, floating sub-heading word, keyword heading word, organism supplementary concept word, protocol supplementary concept word, rare disease supplementary concept word, unique identifier, synonyms, population supplementary concept word, anatomy supplementary concept word] |
| 3 | diet.mp. [mp=title, book title, abstract, original title, name of substance word, subject heading word, floating sub-heading word, keyword heading word, organism supplementary concept word, protocol supplementary concept word, rare disease supplementary concept word, unique identifier, synonyms, population supplementary concept word, anatomy supplementary concept word] |
| 4 | physical activity.mp. [mp=title, book title, abstract, original title, name of substance word, subject heading word, floating sub-heading word, keyword heading word, organism supplementary concept word, protocol supplementary concept word, rare disease supplementary concept word, unique identifier, synonyms, population supplementary concept word, anatomy supplementary concept word] |
| 5 | overweight or obese adults).mp. [mp=title, book title, abstract, original title, name of substance word, subject heading word, floating sub-heading word, keyword heading word, organism supplementary concept word, protocol supplementary concept word, rare disease supplementary concept word, unique identifier, synonyms, population supplementary concept word, anatomy supplementary concept word] |
| 6 | 1 and 2 and 3 and 4 and 5 |

**Table S2.** Proposed search strategy for Embase (Ovid)

| Order | Strategy |
| --- | --- |
| 1 | social support/ or community support/ or family support/ or psychosocial support systems/ or social support.mp.  intervention.mp. or Internet-Based Intervention/ or program*2.mp. |
| 2 | Obesity/ or Overweight/ or Body Weight/ or BMI/ or obesity.mp. or over weight.mp. |
| 3 | diet/ or dietary.mp. [mp=title, abstract, heading word, drug trade name, original title, device manufacturer, drug manufacturer, device trade name, keyword heading word, floating subheading word, candidate term word] |
| 4 | physical activity/ or Exercise/ or sport/ or physical activity.mp. |
| 5 | 1 and 2 and 3 and 4 |

**Table S3.** Proposed search strategy for PsycINFO (Ovid)

| Order | Strategy |
| --- | --- |
| 1 | Obesity/ or Overweight/ or Body Weight/ or BMI.mp. [mp=title, abstract, heading word, table of contents, key concepts, original title, tests & measures, mesh word] |
| 2 | social support/ or community support/ or family support/ or psychosocial support systems/ or social support.mp. [mp=title, abstract, heading word, table of contents, key concepts, original title, tests & measures, mesh word] |
| 3 | diet/ or dietary.mp. [mp=title, abstract, heading word, table of contents, key concepts, original title, tests & measures, mesh word] |
| 4 | physical activity/ or Exercise/ or sport.mp. [mp=title, abstract, heading word, table of contents, key concepts, original title, tests & measures, mesh word] |
| 5 | 1 and 2 and 3 and 4 |

**Table S4.** Proposed search strategy for CINAHL (EBSCO)

| (MH "Obesity") OR (MH "Weight Control") OR (MH "Body Weight") OR (MH "Weight Loss") OR (MH "Weight Reduction Programs") AND (MH "Peer Assistance Programs") OR (MH "Peer Group") OR (MH "Support Groups") OR (MH "Family Support") OR (MH "Support, Social") OR (MH "Community Support") OR (MH "Employment, Supported") OR (MH "Weight Reduction Programs") OR (MH "Social Networking") OR (MH "Intervention Trials") AND (MH "Physical Activity") OR (MH "Diet") |
| --- |

**Table S5.** Proposed search strategy for Web of Science (Core Collection)

| Order | Strategy |
| --- | --- |
| 1 | ALL=(social support) OR ALL=(community support) OR ALL=(family support) or ALL=(peer support) OR ALL=(psychosocial support system) AND ALL=(Intervention) OR ALL=(program*) |
| 2 | ALL=(Obesity or Overweight or Body Weight or BMI or over weight) |
| 3 | ALL=(diet or dietary or nutrition or food consumption) |
| 4 | ALL=(physical activity or exercise or sport) |
| 5 | ALL=(behavio* change) |
| 6 | #5 AND #4 AND #3 AND #2 AND #1 |

**Table S6.** Proposed search strategy for Cochrane Library (Wiley)

| Order | Strategy |
| --- | --- |
| 1 | Obesity, overweight |
| 2 | Social support |
| 3 | intervention, program* |
| 4 | diet |
| 5 | physical activity, sport |
| 6 | 1 and 2 and 3 and 4 and 5 |

**Table S7.** Proposed search strategy for ClinicalTrials.gov

| Order | Strategy |
| --- | --- |
| 1 | Disease = ("obesity" OR "overweight") |
| 2 | Intervention = ("social support" OR family OR peer OR partner OR community) |
| 3 | Result = ("diet" AND "Physical Activity") |
| 4 | AREA[StdAge](ADULT OR OLDER_ADULT) |
| 5 | AREA[HasResults] true |
| 6 | AREA[StdAge](ADULT OR OLDER_ADULT) |
| 7 | 1 and 2 and 3 and 4 and 5 and 6 |

**Table S8.** Proposed search strategy for Google Scholar

| Order | Strategy |
| --- | --- |
| 1 | “obesity” OR “overweight” |
| 2 | “peer support” OR "family support" OR "community-based" |
|  | "intervention" OR "program" |
| 3 | diet AND physical activity |
| 4 | adult |
| 5 | Intitle: 1 and 2 and 3 and 4 and 5 |

**Table S9.** Proposed search strategy for ProQuest

| Order | Strategy |
| --- | --- |
| 1 | ti(obesity OR overweight OR "weight loss") |
| 2 | ab("social support") AND ab(intervention OR trial) |
| 3 | ab("diet" OR "physical activity") |
| 4 | 1 and 2 and 3 |

**Table S10.** Proposed search strategy for PsyArXiv

| Order | Strategy |
| --- | --- |
| 1 | obesity OR overweight |
| 2 | "social support" OR "peer support" |
| 3 | diet OR nutrition OR "physical activity" OR exercise |
| 4 | adult |
| 5 | 1 and 2 and 3 and 4 |
