# Supplementary material for: The Effectiveness of Social Support–Based Interventions for Dietary and Physical Activity in Adults Living With Overweight and Obesity: Protocol for a Systematic Review
Source: JMIR Res Protoc. 2025 Dec 23;14:e81735. doi: 10.2196/81735 (PMC12726821; doi:10.2196/81735)
Supplement: Multimedia Appendix 3 [file resprot-v14-e81735-s003.docx]

#### **Multimedia Appendix 3.** Data extraction categories

#### (1) General information: title, first author, study country (based on study setting), region, and publication year.

#### (2) Study characteristics: study design, inclusion criteria, and number of participants in both intervention and control groups.

#### (3) Participant characteristics: ethnicity, mean age, age range, gender, BMI, and socio-economic status.

#### (4) Intervention details: core components, modality, frequency, primary outcome measures, social support elements, social support measurement tools, key social support activities, facilitators, comparison groups, and follow-up period.

#### (5) Primary outcomes: Measurement tools used to assess dietary and/or physical activity behaviours, including self-report questionnaires, interviewer-administered instruments, and biological measures.

#### (6) Secondary outcomes: Measures of social support (e.g. perceived social support scale), self-efficacy, programme feedback, quality of life, and weight change.

#### (7) Statistical analysis reporting: Reported metrics such as effect sizes, odds ratios, mean differences, p-values, and confidence intervals.
